# Integrative Multiomics and Network Pharmacology Exploration of Active Components and Mechanisms of Action of Qufu Shengxin Ointment in Treating Chronic Nonhealing Wounds

**DOI:** 10.1155/mi/1280142

**Published:** 2026-06-13

**Authors:** Haidong Chen, Yimei Li, Dexuan Chen, Yong Fang, Xuchu Gong, Chaoqun Ma

**Affiliations:** ^1^ Department of General Surgery, Nantong Hospital Affiliated to Nanjing University of Chinese Medicine, Nantong, China, bucm.edu.cn; ^2^ Department of General Surgery, Jiangsu Province Hospital of Chinese Medicine, Affiliated Hospital of Nanjing University of Chinese Medicine, Nanjing, China, njucm.edu.cn

**Keywords:** AKR1B1, autophagy, chronic nonhealing wounds, network pharmacology, PI3K/Akt/mTOR pathway, Qufu Shengxin Ointment, VCAM1

## Abstract

**Background:**

Chronic nonhealing wounds (CNHWs) are characterized by persistent inflammation and impaired autophagy, which hinder normal wound repair. Qufu Shengxin Ointment (QFSO) has shown clinical benefits in treating chronic wounds, but its active components and molecular mechanisms remain largely unclear. This study aimed to investigate the pharmacological mechanisms of QFSO in the treatment of CNHWs.

**Methods:**

Differentially expressed genes (DEGs) were identified from Gene Expression Omnibus (GEO) transcriptomic datasets, and weighted gene co‐expression network analysis (WGCNA) was performed to screen genes associated with CNHWs. Active compounds and potential targets of QFSO were retrieved from the TCMSP database, and a compound–target network was constructed. Mendelian randomization (MR) analysis was applied to evaluate the potential causal effects of key targets on CNHW risk. Gene set enrichment analysis (GSEA) and immune infiltration analysis were conducted to explore biological functions and immune mechanisms. Molecular docking and in vivo animal experiments were performed to validate the predicted interactions and therapeutic effects.

**Results:**

About 1274 DEGs were identified between CNHW and normal wound tissues. Enrichment analyses indicated that the PI3K/Akt/mTOR pathway was significantly involved in CNHW pathogenesis. MR analysis identified AKR1B1 and VCAM1 as potential causal risk factors for CNHWs. Functional enrichment and single‐cell RNA sequencing analyses revealed that these genes participate in immune‐inflammatory regulation and autophagy‐related processes. Molecular docking showed stable binding between key QFSO compounds and the targets AKR1B1 and VCAM1. In vivo experiments demonstrated that QFSO treatment significantly accelerated wound healing. The therapeutic effects were associated with reduced inflammation, enhanced angiogenesis, and activation of autophagy through regulation of the PI3K/Akt/mTOR pathway.

**Conclusions:**

QFSO promotes the repair of CNHWs by regulating the PI3K/Akt/mTOR pathway, enhancing autophagy, alleviating inflammation, and promoting angiogenesis. These findings identify AKR1B1 and VCAM1 as potential molecular targets for the treatment of chronic wounds.

## 1. Introduction

The skin is the largest organ of the human body and plays critical roles in physical protection, immune defense, and maintenance of fluid homeostasis [1, 2]. Various injuries such as trauma, burns, and chronic ulcers can disrupt the integrity of the skin barrier, leading to infection, inflammation, and tissue necrosis [3–5]. Chronic nonhealing wounds (CNHWs) are a particularly challenging clinical condition in which wounds fail to progress through the normal phases of healing despite appropriate medical care [3, 6]. These wounds are typically characterized by delayed granulation tissue formation, impaired angiogenesis, and persistent inflammation, which significantly prolong healing time and increase healthcare costs [7, 8]. With the global aging population and increasing prevalence of chronic diseases such as diabetes and vascular disorders, the incidence of CNHWs continues to rise, posing a substantial clinical and socioeconomic burden [9]. Therefore, understanding the molecular mechanisms underlying CNHWs and identifying effective therapeutic strategies remain critical challenges in wound management.

Autophagy is an evolutionarily conserved intracellular degradation and recycling process that plays an essential role in maintaining cellular homeostasis and adapting to stress conditions [10, 11]. During wound healing, appropriate autophagic activity facilitates the removal of damaged organelles and protein aggregates, thereby reducing oxidative stress and excessive inflammation [12]. In addition, autophagy contributes to the polarization of macrophages toward a reparative phenotype and promotes the proliferation and migration of fibroblasts and endothelial cells, which are essential for granulation tissue formation and neovascularization [12, 13]. Conversely, impaired autophagy results in the accumulation of cellular debris and increased inflammatory signaling, which can disrupt tissue repair and contribute to the development of chronic wounds [14]. Previous studies have shown that modulation of autophagy‐related pathways, including the Beclin‐1, LC3, and mTOR signaling pathways, can significantly influence wound healing outcomes [15–17]. Therefore, targeting autophagy‐related regulatory networks may represent a promising strategy for the treatment of CNHWs.

In recent years, plant‐derived medicines and natural products have attracted increasing attention in wound therapy because of their multitarget pharmacological effects and favorable biocompatibility [18, 19]. Qufu Shengxin Ointment (QFSO) is a traditional Chinese medicinal formulation composed of 16 herbal ingredients, including *Salvia miltiorrhiza* (Danshen), *Angelica sinensis* (Danggui), *Lithospermum erythrorhizon* (Zicao), Myrrha (Moyao), and pearl powder. Clinically, QFSO has shown promising efficacy in the treatment of chronic lower‐extremity ulcers. However, the active components and molecular mechanisms underlying its therapeutic effects remain largely unclear. In the present study, we applied an integrated multiomics and network pharmacology strategy to systematically investigate the pharmacological mechanisms of QFSO in CNHWs. By combining transcriptomic analysis, weighted gene co‐expression network analysis (WGCNA), Mendelian randomization (MR), single‐cell transcriptomics, molecular docking, and in vivo validation, this study aimed to identify key bioactive compounds and molecular targets of QFSO and to elucidate the regulatory mechanisms involved in inflammation, angiogenesis, and autophagy during chronic wound repair.

## 2. Methods

### 2.1. Data Acquisition and Preprocessing

Bulk RNA sequencing data for CNHWs were obtained from the Gene Expression Omnibus (GEO) database. Specifically, the GSE174661 dataset, which includes 10 normal wound samples and five CNHW samples, was used for differential expression analysis. In addition, the single‐cell RNA‐seq dataset GSE265972 was downloaded to explore cell‐type–specific expression patterns of key genes.

In the R environment, the raw expression matrix for GSE174661 was first corrected for batch effects using the sva package and then normalized to ensure comparability across samples [20]. The GSE265972 single‐cell dataset was processed using the Seurat package for quality control, normalization, identification of highly variable genes, principal component analysis (PCA), and UMAP dimensionality reduction and clustering [21].

### 2.2. Differential Expression Analysis

Differential expression between normal and CNHW groups was assessed using the limma package [22]. Genes with |log_2_ fold‐change| >1 and adjusted *p*‐value (adj.*P*) <0.05 were defined as significantly differentially expressed genes (DEGs). Heatmaps and volcano plots were generated to visualize the distribution of up‐ and downregulated genes.

### 2.3. Gene Ontology (GO) and KEGG Enrichment Analysis

To explore the biological roles and pathways associated with the DEGs, GO enrichment (for molecular function [MF], cellular component, and biological process [BP]) and KEGG pathway analysis were performed sequentially using the clusterProfiler package [23]. This identified key biochemical pathways potentially involved in CNHW pathogenesis.

### 2.4. WGCNA

The WGCNA R package was employed to construct a weighted co‐expression network and identify modules of co‐expressed genes associated with CNHWs [24]. After preprocessing and outlier removal, a correlation matrix was constructed and transformed into an adjacency matrix using an optimal soft‐threshold power. From the adjacency matrix, a topological overlap matrix (TOM) was derived. Genes were then clustered into modules by average linkage hierarchical clustering of the TOM‐based dissimilarity measure. The module most strongly correlated with the chronic nonhealing phenotype was selected for downstream analysis.

### 2.5. Collection of QFSO Active Compounds and Target Screening

Candidate bioactive compounds in QFSO were identified from the TCMSP database (https://tcmsp-e.com/tcmsp.php) using oral bioavailability ≥30% and drug‐likeness ≥0.18 as cutoffs [25]. For each compound, its predicted protein targets were retrieved, deduplicated, and compiled into a target gene list. Cytoscape 3.7.2 was then used to construct and visualize the compound–target interaction network, providing a framework for subsequent network topology and functional analyses.

### 2.6. MR Analysis

To assess the causal effects of core target genes on CNHW risk, two‐sample MR was performed using the TwoSampleMR *R* package [26]. SNPs significantly associated with each gene’s expression (*p* < 5 *×* 10^−8^) were extracted from public eQTL (IEU Open GWAS) and pQTL (deCODE Genetics) summary statistics. Linkage disequilibrium clumping (*R* ^2^ < 0.01, 10 Mb window) was applied to ensure the independence of instruments. Outcome summary data were obtained from the FinnGen CNHWs GWAS [27]. The inverse‐variance weighted (IVW) method estimated causal effects (significance threshold *p* < 0.05). Heterogeneity was evaluated by Cochran’s *Q* test, and leave‐one‐out sensitivity analyses ensured the robustness of causal inference. A detailed description of the MR reporting items is provided in Supporting Information 1: Table S3. Horizontal pleiotropy was evaluated using the MR‐Egger intercept test, where a nonsignificant intercept indicates the absence of directional pleiotropy. Heterogeneity among instrumental variables was assessed using Cochran’s *Q* test. In addition, leave‐one‐out sensitivity analysis was conducted to evaluate whether the causal estimates were driven by any single SNP.

### 2.7. Immune Infiltration Analysis

The CIBERSORT algorithm was applied to deconvolute the GSE174661 bulk RNA‐seq profiles, estimating relative abundances of 22 immune cell types in normal versus CNHW samples [28]. Only samples with CIBERSORT *p* < 0.05 were retained. Spearman correlation tests then assessed associations between inferred immune cell proportions and core target gene expression, revealing the targets’ potential roles in the wound‐immune microenvironment.

### 2.8. Gene Set Enrichment Analysis (GSEA)

GSEA was conducted with the clusterProfiler package to identify pathways differentially enriched between high‐ and low‐expression groups for candidate genes (*p* < 0.05) [29]. Enrichplot was used for visualization. Additionally, unsupervised gene set variation analysis (GSVA) was performed with the GSVA package to quantify relative enrichment of pathways across individual samples [30].

### 2.9. Single‐Cell RNA‐Seq Data Analysis

Single‐cell RNA‐seq data were processed in Seurat, as described in Section 2.1 [31]. UMAP plots and violin plots were generated to examine expression differences of AKR1B1 and VCAM1 across annotated cell types, elucidating their cell‐specific roles in CNHWs.

### 2.10. Molecular Docking

Two‐dimensional structures of candidate ligands were downloaded from PubChem and converted to three‐dimensional mol^2^ files in ChemOffice [32]. High‐resolution crystal structures of target proteins were obtained from the RCSB PDB and prepared in PyMOL by removing water molecules and ligands and then saved as PDB files [33]. AutoDock Vina 1.5.6 performed docking using grid boxes defined around the binding pockets [34]. Protein receptors and ligands were prepared with AutoDock Tools (adding hydrogens and assigning torsions). The best binding pose (lowest binding energy) was selected for each ligand–receptor pair. Interactions were visualized in PyMOL and Discovery Studio 2019 to produce 2D interaction diagrams and 3D conformations. Binding energies < –5.0 kcal/mol indicate good affinity and < –7.0 kcal/mol indicates strong binding.

### 2.11. Animal Experiments

About 100 12‐week‐old male Sprague–Dawley rats (340–420 g) were acclimated for 1 week under SPF conditions (approved by the Nantong University Animal Care and Use Committee). After 24 h of fasting with water ad libitum, rats were anesthetized with ketamine (90 mg/kg) and xylazine (9 mg/kg) intraperitoneally. Dorsal fur was shaved and disinfected, and then a 20 mm diameter full‐thickness skin defect was created down to the fascia. Immediately postsurgery, rats received intramuscular hydrocortisone acetate (8 mg/100 g) to delay healing, and penicillin potassium (4000 U/rat) was administered daily for 4 days to prevent infection. Successful model induction was confirmed by H&E staining and pathogen culture.

Ninety‐eight rats with stable wounds were randomized into four groups (*n* = 20 each; blank control *n* = 18 with only saline dressing): model, Fujifu (5 g gel), MEBO (60 g ointment), and QFSO (20 g). From day 1 postmodeling, treatment groups received daily wound cleaning with a furacin solution (1:5000), followed by application of drug‐impregnated gauze strips at 0.2 g/cm^2^, covered with sterile gauze and tape. The model group received saline only.

On days 3, 7, and 14, six rats per group (plus six blank controls; two extras reserved) were euthanized by an intraperitoneal injection of an overdose of pentobarbital sodium (150 mg/kg), and the granulation tissue plus surrounding normal skin (0.5 cm margin) was harvested. To eliminate the environmental concerns associated with pentobarbital residues, all remaining animal carcasses were safely disposed of via alkaline hydrolysis. This modern, eco‐friendly method completely degrades the euthanizing agent without generating any harmful emissions. Samples were split: one half fixed in 4% paraformaldehyde for H&E and immunohistochemistry (PI3K, AKT, mTOR, HIF‐1α, VEGF, TNF‐α, ULK1, LC3‐II, and Beclin‐1); the other half was snap‐frozen in liquid nitrogen at –80°C for RT‐qPCR (HIF‐1α, VEGF, TNF‐α, ULK1, LC3‐II, Beclin‐1; GAPDH reference; Supporting Information 2: Table S1).

The animal experiment was approved by the Laboratory Animal Center of NTU (Grant 20220510‐003). The ARRIVE Essential 10 and Recommended Set checklists are completed and provided as Supporting Information 3: Table S2.

### 2.12. Statistical Analysis

All analyses were performed in R. Data are presented as mean ± SD. One‐way ANOVA followed by Tukey’s post hoc test determined group differences (*p* < 0.05). Continuous variables were compared by the Wilcoxon rank‐sum test and categorical variables by the chi‐square test. The false discovery rate (FDR) correction adjusted for multiple testing to control type I error.

### 2.13. Study Design Overview

**Figure 1:**
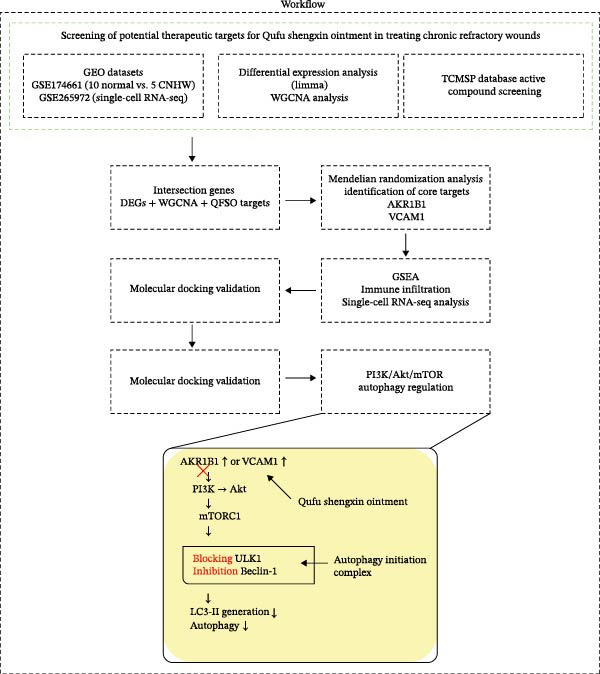
Schematic workflow of this study, illustrating the complete sequence of analyses: data collection, differential gene screening, intersection with WGCNA modules, Mendelian randomization causal analysis, functional enrichment and immune infiltration analysis, followed by single‐cell lineage validation, molecular docking, and in vivo animal model verification.

The overall study workflow, including transcriptomic analysis, network pharmacology screening, MR analysis, immune infiltration analysis, single‐cell transcriptomic validation, molecular docking, and in vivo experimental validation, is summarized in Figure 1.

## 3. Results

### 3.1. Screening of CNHW‐Related Genes

Using the limma package to compare CNHW samples with normal controls in the GSE174661 dataset, we identified 1274 DEGs, of which 594 were upregulated and 680 were downregulated (Figure 2A,B). We then constructed a weighted gene co‐expression network via WGCNA and found that the yellow module (MEyellow) exhibited the highest correlation with the nonhealing phenotype (*r* = 0.77, *p* = 9 × 10^−4^; Figure 2C). Intersection of the MEyellow genes with the DEGs yielded 486 candidate genes (Figure 2D).

Figure 2Screening of CNHW‐related genes. (A) Heatmap of DEGs in GSE174661. (B) Volcano plot of DEGs (green, downregulated; red, upregulated). (C) WGCNA module–trait correlation heatmap showing association with CNHW phenotype. (D) Venn diagram showing 486 candidate genes at the intersection. (E) GO biological process enrichment of candidate genes. (F) KEGG pathway enrichment of candidate genes.

**Figure:**
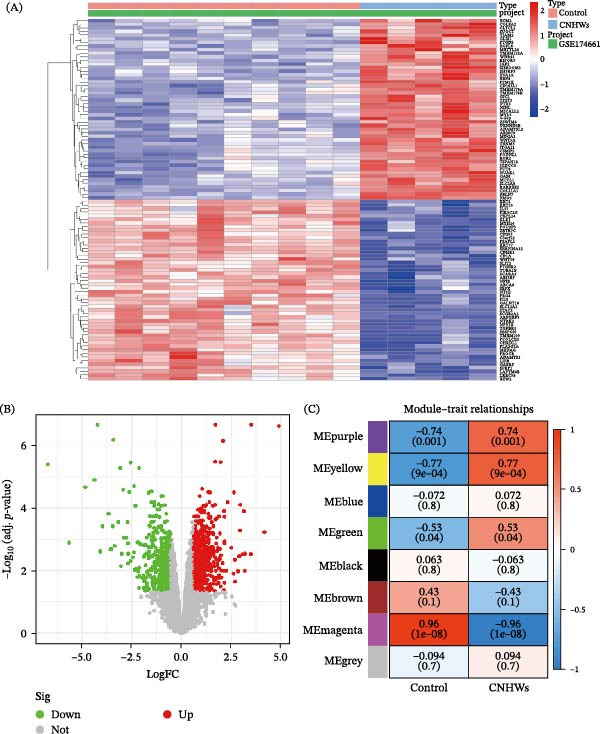

**Figure:**
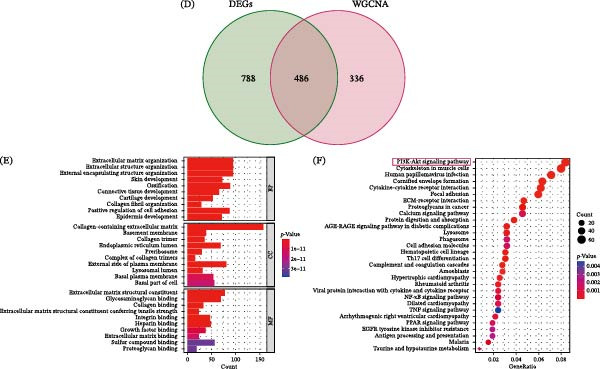

GO enrichment analysis of the 486 candidate genes revealed significant enrichment in BPs related to tissue development and extracellular matrix organization. The top enriched BP terms included epidermis development, positive regulation of cell adhesion, collagen fibril organization, connective tissue development, skin development, extracellular matrix organization, extracellular structure organization, cartilage development, and ossification. In the MF category, the genes were mainly enriched in extracellular matrix structural constituent, collagen binding, integrin binding, extracellular matrix binding, growth factor binding, proteoglycan binding, glycosaminoglycan binding, sulfur compound binding, and heparin binding (Figure 2E, Supporting Information 4: Figure S2A).

KEGG pathway analysis further identified several significantly enriched pathways, including the PI3K–Akt signaling pathway, cytokine–cytokine receptor interaction, focal adhesion, ECM–receptor interaction, calcium signaling pathway, complement and coagulation cascades, NF‐κB signaling pathway, TNF signaling pathway, and cell adhesion molecules (CAMs) (Figure 2F). Among these pathways, the PI3K–Akt signaling pathway contained the largest number of candidate genes (*n* = 66). Enrichment analysis showed that multiple genes were significantly enriched in the PI3K–Akt signaling pathway and autophagy‐related BPs.

### 3.2. MR Analysis Reveals Causal Associations Between Key Genes and CNHW Risk

**Figure 3:**
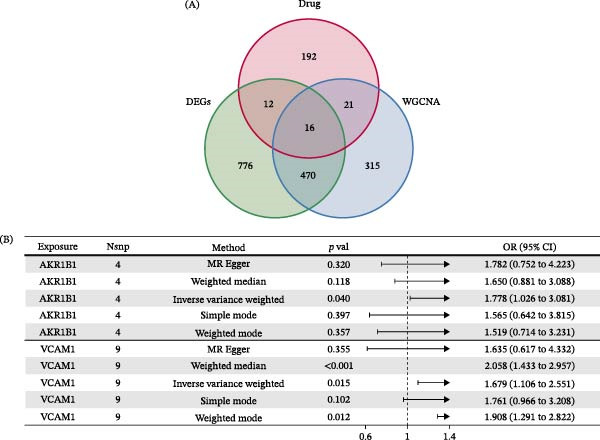
Mendelian randomization analysis revealing causal associations between key genes and CNHW risk. (A) Venn diagram of WGCNA modules, DEGs, and QFSO active compound targets. (B) MR scatter plots and causal effect summary for AKR1B1 and VCAM1.

From the TCMSP database, 172 active compounds and their 241 predicted targets were obtained. Intersection with the WGCNA MEyellow and DEGs yielded 16 candidate genes (Figure 3A). Two‐sample MR analysis using the TwoSampleMR package showed that the expression of AKR1B1 and VCAM1 is significantly positively associated with the risk of CNHWs (Figure 3B).

For AKR1B1, the MR scatter plot (Supporting Information 4: Figure S2B) indicates a positive *β* value for each additional effect allele, with IVW estimates consistent with MR‐Egger, weighted median, and mode‐based methods. The forest plot (Supporting Information 4: Figure S2C) shows that the vast majority of SNPs exert positive effects; Cochran’s *Q* test indicates no significant heterogeneity (*p* > 0.1). The funnel plot (Supporting Information 4: Figure S2D) appears approximately symmetric, and leave‐one‐out sensitivity analysis (Supporting Information 4: Figure S2E) did not identify any single SNP driving the overall association.

Similarly, for VCAM1, the MR scatter plot (Supporting Information 4: Figure S2F) reveals a positive association between effect alleles and nonhealing wound risk. The forest plot (Supporting Information 4: Figure S2G) shows consistent effect directions across SNPs, the funnel plot (Supporting Information 4: Figure S2H) remains symmetric, and leave‐one‐out analysis (Supporting Information 4: Figure S2I) confirms robustness, with no single SNP substantially altering the overall estimate.

Sensitivity analyses showed no evidence of significant horizontal pleiotropy based on the MR‐Egger intercept test (*p* > 0.05). Cochran’s *Q* test indicated no substantial heterogeneity among the instrumental variables. Furthermore, leave‐one‐out analysis demonstrated that the causal estimates were not driven by any single SNP.

### 3.3. Molecular Characteristics of AKR1B1 and VCAM1 in CNHWs

**Figure 4:**
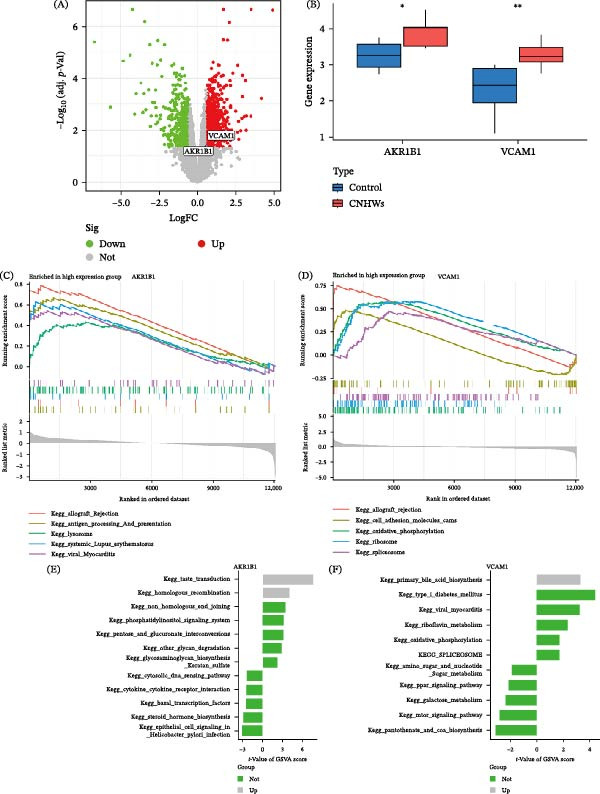
Molecular features of AKR1B1 and VCAM1 in CNHWs. (A) Localization of both genes on the DEG volcano plot. (B) Boxplots comparing gene expression in normal versus CNHW tissues. (C, D) GSEA enrichment curves for AKR1B1 and VCAM1. (E, F) GSVA heatmaps showing pathway enrichment scores for both genes.

Both AKR1B1 and VCAM1 are markedly upregulated in the CNHW tissue. On the volcano plot of DEGs, they localize in the upregulated region (Figure 4A), and boxplots quantitatively confirm that median expression levels of AKR1B1 and VCAM1 are significantly higher in the CNHW group than in the controls (Figure 4B).

To elucidate their functional networks, we performed GSEA. Samples with high AKR1B1 expression are enriched for T/B cell activation, autoimmune‐related mechanisms, and the autophagy–lysosome pathway, indicating a central role in immune‐inflammatory regulation and cellular clearance processes (Figure 4C). In contrast, high VCAM1 expression samples show significant enrichment in allograft rejection, CAMs, oxidative phosphorylation, ribosome, and spliceosome pathways, reflecting VCAM1’s key involvement in enhancing cell–cell/cell–matrix adhesion and metabolic reprograming (Figure 4D).

GSVA analysis further demonstrated that the high AKR1B1 expression subgroup scores higher in gene sets related to extracellular matrix metabolism, signal transduction, and genome maintenance, whereas the high VCAM1 expression subgroup exhibits significantly elevated scores in protein biosynthesis, energy metabolism, and specific immunopathological processes (Figure 4E,F).

### 3.4. Immune Cell Infiltration Analysis in CNHW Tissues

Immune cell infiltration patterns differ between normal and CNHW tissues (Figure 5A). Correlation analysis reveals complex interrelationships among immune cell types (Figure 5B). Notably, CD8^+^ T‐cell abundance is increased in CNHWs, whereas activated dendritic cell (DC) levels are decreased (Figure 5C). Elevated AKR1B1 expression correlates with increased proportions of unpolarized macrophages (M0) and follicular helper T cells (Tfh) (Figure 5D,E), while VCAM1 expression shows no significant correlation with any immune cell subset (Figure 5F).

Figure 5Immune cell infiltration analysis in CNHW tissues. (A) Bar plots of relative abundance of 22 immune cell types in normal versus CNHW samples. (B) Correlation matrix among immune cell types. (C) Boxplots comparing immune cell abundances between groups. (D) Correlation heatmap of AKR1B1 and VCAM1 with immune cells. (E, F) Plots showing regulation of immune cell infiltration by AKR1B1 and VCAM1 in CNHW samples.

**Figure:**
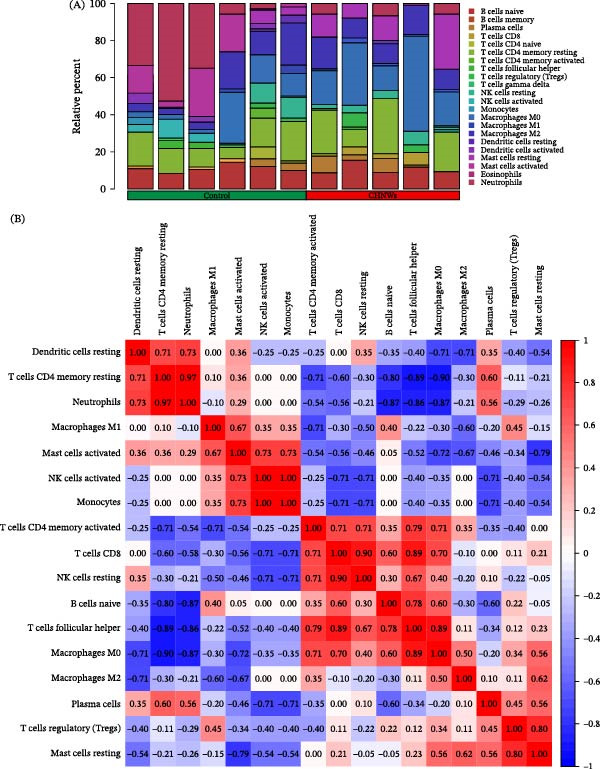

**Figure:**
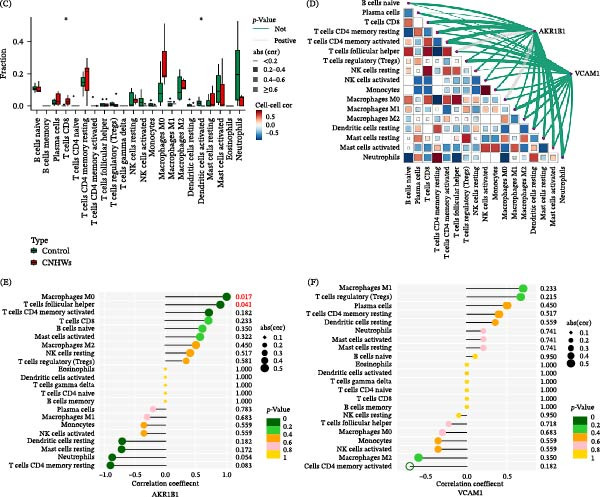

### 3.5. Single‐Cell Transcriptomic Analysis Reveals Cell‐Type–Specific Expression of Key Genes

**Figure 6:**
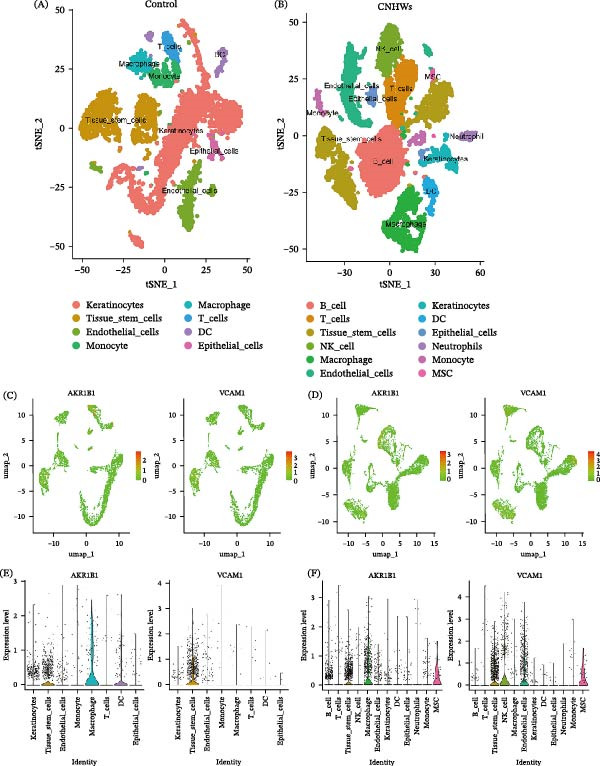
Single‐cell transcriptome analysis revealing cell‐type–specific expression of key genes. (A, B) Cell‐type annotation of the identified clusters in control and CNHW samples. (C, D) UMAP feature plots showing the expression distribution of AKR1B1 and VCAM1 in control and CNHW samples. (E, F) Violin plots showing the expression levels of AKR1B1 and VCAM1 across different cell types in control and CNHW samples.

In normal wound samples, UMAP dimensionality reduction and clustering clearly delineated major cell subpopulations, including tissue stem cells, macrophages, and DCs (Figure 6A,B; Supporting Information 4: Figure S2J,K). AKR1B1 shows moderate to high expression across these stem cell, macrophage, and DC clusters, while its expression is relatively low in other cell types, suggesting a foundational role in immune surveillance and homeostasis in healthy wound tissues. In contrast, VCAM1 expression is largely confined to the tissue stem cell cluster and at relatively low levels, reflecting its maintenance of a basal surveillance state in healthy skin.

By comparison, UMAP clustering of CNHW samples (Figure 6C,D) reveals a similar cellular composition but a marked expansion of the AKR1B1 expression landscape. AKR1B1 maintains high expression in tissue stem cells and macrophages and is notably enriched in mesenchymal stem cells (MSCs) (Figure 6E,F), indicating broader regulation of stem cell metabolism and immune responses in chronic wounds. VCAM1 is also significantly upregulated in CNHW samples; beyond tissue stem cells, it is highly expressed in natural killer (NK) cells, endothelial cells, and MSC clusters (Figure 6D,F), consistent with endothelial activation and increased leukocyte adhesion driven by persistent inflammation.

### 3.6. Molecular Docking Results of Core Targets and Core Compounds

In molecular docking experiments, all selected natural active compounds demonstrated favorable binding affinities to AKR1B1 and VCAM1, with binding energies ranging from –6.1 to –12.4 kcal/mol. This indicates that they can stably occupy the receptor binding pockets and form diverse molecular interactions.

For AKR1B1, digallate exhibited the lowest binding energy (–12.4 kcal/mol). It is anchored by van der Waals interactions with residues ASN162, PRO130, PHE311, and LEU190 and by hydrogen bonds with HIS163, GLN192, GLU193, and ASN292. Additional hydrophobic contacts with LEU195 and LYS194 further stabilize the ligand–receptor complex (Figure 7A). Quercetin also binds tightly to AKR1B1 with a binding energy of –12.4 kcal/mol, primarily via van der Waals contacts with PHE311, PRO310, and LEU190 and hydrogen bonds involving ARG296, ASN294, THR191, and GLU193 (Figure 7C). Isorhamnetin binds AKR1B1 at –11.6 kcal/mol and stigmasterol at –11.3 kcal/mol; both interactions are stabilized by a network of van der Waals forces and hydrogen bonds, confirming the high affinity of flavonoids and sterols for AKR1B1 (Figure 7B,D).

Figure 7Molecular docking results of core targets and core compounds. (A) Docking of quercetin with AKR1B1. (B) Docking of digallate with AKR1B1. (C) Docking of stigmasterol with AKR1B1. (D) Docking of isorhamnetin with AKR1B1. (E) Docking of quercetin with VCAM1. (F) Docking of kaempferol with VCAM1.

**Figure:**
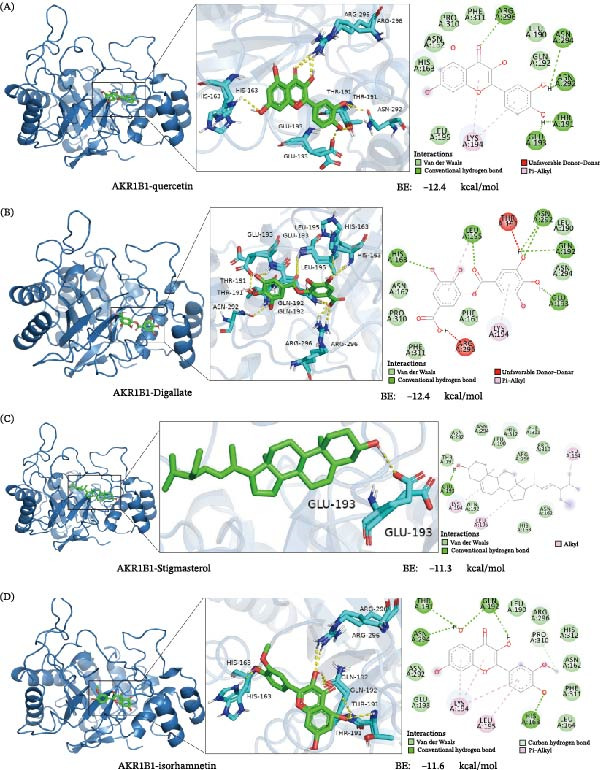

**Figure:**
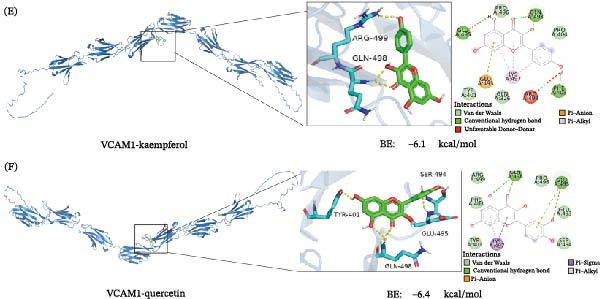

In docking with VCAM1, quercetin showed a binding energy of –6.4 kcal/mol, stabilized by van der Waals interactions with ARG499, PHE403, and TYR401, as well as by hydrogen bonds and π–anion interactions with GLN498 and GLU495 (Figure 7E). Kaempferol interacted with VCAM1 at –6.1 kcal/mol, with its binding supported by van der Waals forces, hydrogen bonds, and minor hydrophobic contacts (Figure 7F).

### 3.7. In Vivo Evaluation of QFSO in a Rat Model of CNHWs

In the rat model of CNHWs, QFSO markedly accelerated wound closure. Compared with the model group, the QFSO, Fujifu, and MEBO treatment groups all exhibited faster wound contraction at days 3, 7, and 14 postoperation (Figure 8A). H&E staining showed that the model group maintained heavy inflammatory cell infiltration, sparse and loose granulation tissue, and impaired re‐epithelialization and collagen remodeling. In contrast, the QFSO group displayed a significant reduction in inflammatory cells from day 3, thicker and more vascularized granulation tissue with new epidermis at day 7, and by day 14 a continuous epidermis with densely arranged collagen fibers—outperforming both Fujifu and MEBO groups (Figure 8B).

Figure 8In vivo study of QFSO intervention in CNHW rat model. (A) Comparison of wound closure among groups at different time points. (B) H&E staining of wound tissues at corresponding time points. (C) Protein expression levels of Beclin‐1, LC3‐II, ULK1, HIF‐1α, TNF‐α, and VEGF after QFSO treatment. Data are presented as mean ± SD. Statistical significance is indicated by horizontal brackets connecting the compared groups.  ^∗^ *p* < 0.05,  ^∗∗^ *p* < 0.01.

**Figure:**
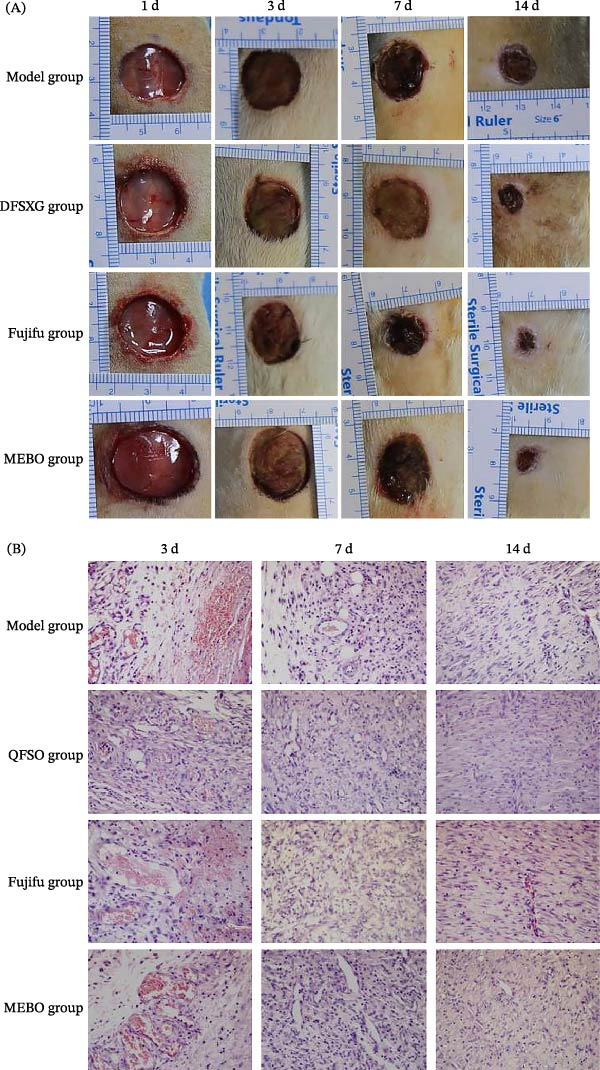

**Figure:**
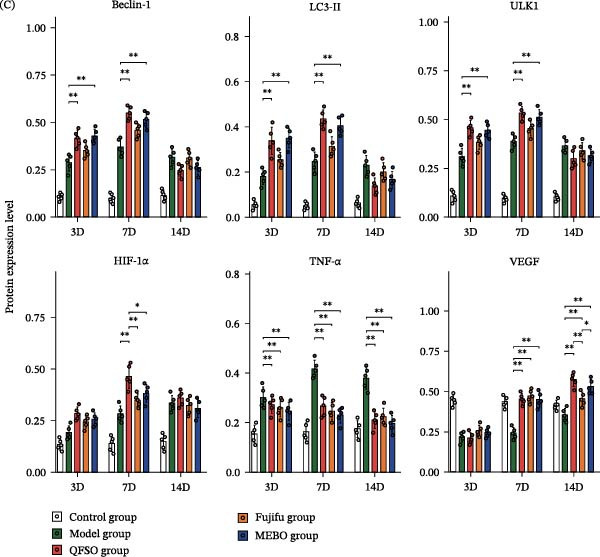

At the mRNA level, ULK1, Beclin‐1, and LC3‐II were significantly upregulated in the QFSO group compared with the model group, with expression comparable to that of MEBO and no obvious changes in the Fujifu group. HIF‐1α expression in the QFSO group was significantly higher than in the other groups at day 7. TNF‐α levels were significantly reduced in the QFSO, Fujifu, and MEBO groups versus the model group. VEGF expression in all three treatment groups was higher than the model at day 7 but lower than that in the model at day 14 (Supporting Information 4: Figure S2L).

At the protein level, ULK1, Beclin‐1, and LC3‐II were significantly elevated in the QFSO group at days 3 and 7, mirroring the MEBO trend, while the Fujifu group showed only modest increases. HIF‐1α in the QFSO group peaked significantly at day 7. TNF‐α was significantly reduced in all three treatment groups compared to the model. VEGF levels in QFSO, Fujifu, and MEBO groups were higher than those in the model at days 7 and 14, with QFSO and MEBO showing higher VEGF than Fujifu at day 14 (Figure 8C).

Immunohistochemistry further demonstrated that PI3K, AKT, and mTOR staining intensities remained high in the model group at days 3, 7, and 14, indicating persistent pathway activation postinjury. QFSO produced the strongest inhibition of the PI3K/Akt/mTOR axis, with MEBO also reducing signaling at each time point, albeit to a lesser extent (Supporting Information 5: Figure S1A–C). Autophagy markers Beclin‐1 and LC3‐II were weakly stained in the model group, indicating impaired autophagic activity; both markers increased in the QFSO group at days 7 and 14, with MEBO showing moderate upregulation (Supporting Information 5: Figure S1D,E). Additionally, IL‐8 in the QFSO group dropped sharply by day 3, effectively curbing early inflammation. HIF‐1α exhibited a transient peak at day 7 in the QFSO group, reflecting its proangiogenic activation (Supporting Information 5: Figure S1G). These findings indicate that QFSO not only promptly alleviates chronic hypoxia and inflammation but also facilitates a smooth transition from inflammation to the regeneration and remodeling stages.

## 4. Discussion

Despite adequate conventional treatment, CNHWs often remain trapped in the inflammatory phase due to persistent inflammation and autophagy dysfunction, failing to progress into the proliferative and remodeling stages [35–37]. Autophagy plays a central role in clearing damaged organelles, inhibiting excessive inflammation, and promoting macrophage polarization toward a repair phenotype [38, 39]. By integrating multiomics analyses with animal model data, our study elucidates the molecular mechanism by which QFSO restores autophagic activity to treat CNHWs. Our integrated multiomics analysis identified AKR1B1 and VCAM1 as key targets associated with CNHWs. Functional enrichment and MR analyses consistently indicated significant involvement of the PI3K–Akt signaling pathway and autophagy‐related BPs. These findings suggest that the PI3K–Akt–mTOR–autophagy axis may play a central regulatory role in the therapeutic effects of QFSO during chronic wound repair.

Following differential expression analysis and WGCNA module selection, KEGG enrichment identified the PI3K–Akt signaling pathway as the most enriched in CNHW tissue, followed by CAMs, lysosome, and TNF signaling pathways. This indicates that the PI3K/Akt axis plays a pivotal role in maintaining the inflammatory microenvironment of chronic wounds and in promoting cell survival and proliferation [40]. From a biological perspective, PI3K activation phosphorylates Akt, which in turn activates mTORC1, which in turn phosphorylates and inhibits ULK1 and Beclin‐1, thereby negatively regulating the autophagy initiation complex [41, 42]. Our immunohistochemistry and RT‐qPCR data both show markedly reduced ULK1, Beclin‐1, and LC3‐II levels in the model group, confirming concurrent PI3K/Akt/mTOR overactivation and autophagy suppression. Impaired autophagy leads to accumulation of dysfunctional organelles and protein debris, exacerbating oxidative stress and inflammatory mediator release, thereby hindering transition into the proliferative and remodeling phases. Thus, restoring autophagic function and inhibiting PI3K/Akt/mTOR hyperactivation are crucial strategies to break the pathological cycle of CNHWs and promote wound healing.

Through TCMSP screening, 16 candidate genes were identified by intersecting QFSO’s active compounds’ targets with WGCNA modules and DEGs. MR analysis further confirmed that AKR1B1 and VCAM1 expression are significantly positively associated with CNHW risk. AKR1B1 encodes aldose reductase, which catalyzes the reduction of aldoses to generate reactive oxygen species (ROS), triggering oxidative stress and thereby activating the PI3K/Akt pathway [43, 44]. Sustained Akt phosphorylation enhances mTORC1 activity, which in turn phosphorylates and inhibits ULK1 and Beclin‐1, blocking the assembly and function of the autophagy initiation complex.

Diabetic patients often develop CNHWs [45, 46], and studies have shown that AKR1B1 plays a key role in the occurrence of diabetic complications [47]. Therefore, targeting AKR1B1 and developing its active inhibitors are expected to provide new intervention strategies for the treatment of diabetes and CNHWs. VCAM1, an endothelial CAM, contains PI3K/Akt–responsive elements in its promoter and is upregulated by Akt‐activated transcription factors such as NF‐κB, forming a positive feedback loop that further enhances inflammatory cell infiltration and adhesion [48, 49]. VCAM1 overexpression not only exacerbates sustained inflammation in the wound but also indirectly activates mTORC1 via the PI3K/Akt pathway, thereby inhibiting autophagy.

Autophagy is critical for clearing damaged organelles, suppressing excessive inflammation, and promoting the functional transition of repair cells [50]. In this study, AKR1B1 and VCAM1 converge on the PI3K/Akt/mTOR axis through dual mechanisms, leading to suppression of the autophagy initiation complex, blockade of ULK1/Beclin‐1 signaling, and reduction of LC3‐II levels, thus interrupting autophagy‐mediated cellular clearance. This autophagy deficiency both intensifies oxidative stress and inflammation and weakens fibroblast and endothelial cell migration and differentiation, forming the molecular basis of CNHWs.

AKR1B1’s pronounced overexpression in CNHWs promotes oxidative stress metabolism via the aldose reductase pathway and participates in pathology through multiple immune and autophagy‐related pathways. In contrast, VCAM1 overexpression focuses on adhesion and metabolic reprograming. Overall, AKR1B1 and VCAM1 establish a chronic wound microenvironment characterized by low autophagy, high inflammation, and metabolic imbalance via complementary autophagy/immune regulation and adhesion/metabolic reprograming pathways, driving the nonhealing pathological cycle. High AKR1B1 expression also correlates with increased M0 and Tfh, indicating stalled macrophage differentiation and aberrant humoral immunity in CNHWs. Single‐cell profiling shows AKR1B1 highly expressed across multiple reparative and inflammatory lineages—tissue stem cells, macrophages, and MSCs—whereas in normal tissue, its expression is limited to tissue stem cells, macrophages, and DCs at low levels, reflecting its basal role in redox homeostasis. Similarly, VCAM1 is broadly upregulated in CNHW samples, including in tissue stem cells, NK cells, endothelial cells, and MSCs, consistent with endothelial activation and leukocyte adhesion under persistent inflammation. By contrast, normal samples exhibit minimal VCAM1, confined to a subset of tissue stem cells. These data indicate that CNHWs trigger co‐overexpression of AKR1B1 and VCAM1 in reparative and immune compartments, correlating with PI3K–Akt activation, mTORC1‐mediated ULK1/Beclin‐1 suppression, and LC3‐II–driven autophagy reduction, thereby impeding normal wound resolution. Targeting AKR1B1 and VCAM1 to inhibit the PI3K/Akt/mTOR pathway may restore autophagy and accelerate transition into the proliferative and remodeling phases. Molecular docking confirms that QFSO’s key compounds bind with high affinity to functional sites on AKR1B1 and VCAM1 at the atomic level, providing structural support for their potential roles in inhibiting PI3K/Akt/mTOR signaling, restoring autophagy, and alleviating inflammation.

In the CNHW rat model, QFSO significantly accelerated wound healing and, by multiple readouts, validated its mechanism of inhibiting the PI3K/Akt/mTOR pathway while activating autophagy. At days 3, 7, and 14, the QFSO group exhibited significantly faster wound closure than the model group, comparable to that of the Fujifu and MEBO groups. H&E staining further confirmed that inflammatory cell infiltration in the QFSO group was markedly reduced as early as day 3; by day 7, the granulation tissue was denser and well organized; and by day 14, the epidermis was intact with orderly collagen fiber alignment. In contrast, the model group showed persistent inflammation, sparse granulation tissue, and a pronounced delay in the remodeling phase. These findings demonstrate that QFSO rapidly shifts the chronic wound environment from a prolonged inflammatory state into the proliferative and remodeling phases.

MEBO has been shown to activate autophagy in diabetic ulcers [51]. RT‐qPCR and immunohistochemistry revealed that both mRNA and protein levels of ULK1, Beclin‐1, and LC3‐II in the QFSO group were significantly higher than in the model group at days 3 and 7—trending similarly but more robustly than MEBO and exceeding the modest increases seen with Fujifu. This enhancement of autophagic activity aligns with the early need to clear necrotic cells and protein aggregates, creating a cleaner wound microenvironment that fosters fibroblast and endothelial cell migration and proliferation. Additionally, TNF‐α and IL‐8 expression in the QFSO group dropped sharply by day 3, mitigating early inflammatory stimuli. HIF‐1α and VEGF were moderately upregulated at day 7, promoting neovascularization and alleviating wound hypoxia. This temporal coordination of autophagy activation and angiogenesis facilitates a swift transition into the proliferative phase once debris clearance is achieved. Therefore, the in vivo results fully support our in vitro and bioinformatics predictions: QFSO improves CNHW healing by inhibiting PI3K/Akt/mTOR to relieve negative regulation of ULK1/Beclin‐1–mediated autophagy, enhancing autophagic flux, while simultaneously coordinating inflammation resolution and angiogenesis.

## 5. Conclusion

Through an integrated multiomics and network pharmacology approach—combined with MR, single‐cell transcriptomics, molecular docking, and animal experiments—this study elucidates the key mechanisms by which QFSO promotes repair of CNHWs at multiple levels. Differential expression analysis and WGCNA pinpointed the PI3K/Akt/mTOR pathway as the central network driving chronic inflammation and autophagy suppression in nonhealing wounds. MR established the causal roles of AKR1B1 and VCAM1 within this network, revealing their molecular signatures in the autophagy–lysosome pathway, immune inflammation, and metabolic reprograming. Single‐cell analysis precisely localized high expression of these genes in tissue stem cells, macrophages, MSC, and endothelial cells—critical lineages involved in repair and inflammation. Molecular docking demonstrated that natural compounds such as digallate and quercetin bind with high affinity to AKR1B1 and VCAM1, providing a structural basis for targeted inhibition. In the rat model, QFSO effectively suppressed PI3K/Akt/mTOR signaling to relieve negative regulation of ULK1/Beclin‐1/LC3‐II–mediated autophagy while coordinating inflammation resolution and angiogenesis, thereby significantly accelerating transition from the inflammatory to proliferative and remodeling phases. In summary, this work not only furnishes systematic molecular evidence for the modernization and clinical translation of QFSO but also lays a solid foundation for precision treatment strategies and novel drug target development in chronic wound management.

**Nomenclature**
AKR1B1: Aldo‐keto reductase family 1 member B1
CNHWs: Chronic nonhealing wounds
DEGs: Differentially expressed genes
HIF‐1α: Hypoxia‐inducible factor 1 alpha
IHC: Immunohistochemistry
IL‐8: Interleukin‐8
KEGG: Kyoto Encyclopedia of Genes and Genomes
LC3‐II: Microtubule‐associated protein 1 light chain 3 beta (lipidated form)
MR: Mendelian randomization
PDB: Protein Data Bank
pQTL: Protein quantitative trait loci
QFSO: Qufu Shengxin Ointment
TCMSP: Traditional Chinese Medicine Systems Pharmacology database
TNF‐α: Tumor necrosis factor alpha
VEGF: Vascular endothelial growth factor
WGCNA: Weighted gene co‐expression network analysis.

## Author Contributions

**Haidong Chen**: conceptualization, formal analysis, methodology, writing – original draft. **Yimei Li**: conceptualization, data curation, investigation, software, writing – original draft. **Dexuan Chen**: data curation, formal analysis, resources, validation, writing – review and editing. **Yong Fang**: data curation, formal analysis, resources, visualization, writing – original draft. **Xuchu Gong**: data curation, investigation, software, writing – review and editing. **Chaoqun Ma**: funding acquisition, methodology, project administration, supervision, writing – review and editing.

## Funding

This work is supported by the Research Project of Jiangsu Society of Traditional Chinese Medicine (Grant PDJH2024051), the Key Project of Jiangsu Province Institute of Traditional Chinese Medicine School of Thought Research (Grant LPZD2025009), the Nantong Science and Technology Bureau Social and People’s Livelihood Science and Technology Program (Grant MSZ2025183), the Nantong Municipal Health Commission General Project (Grant MS2024042), the Seventh Batch of National Famous Old TCM Teacher Training Program (National TCM People’s Education Letter [2022]76), and the Natural Science Foundation of Nanjing University of Chinese Medicine (Grant XZR2021084).

## Disclosure

All claims expressed in this article are solely those of the authors and do not necessarily represent those of their affiliated organizations, or those of the publisher, the editors, and the reviewers. Any product that may be evaluated in this article or claim that may be made by its manufacturer is not guaranteed or endorsed by the publisher. All authors read and approved the final manuscript.

## Ethics Statement

The animal experiment was approved by the Laboratory Animal Center of NTU (Number 20220510‐003).

## Conflicts of Interest

The authors declare no conflicts of interest.

## Supporting Information

Additional supporting information can be found online in the Supporting Information section.

## Supporting information


**Supporting Information 1** Table S3: STROBE‐MR checklist of recommended items to address in reports of Mendelian randomization studies.

**Supporting Information 2** Table S1: Primer sequences used for RT‐qPCR in this study.

**Supporting Information 3** Table S2: ARRIVE guidelines 2.0: author checklist. (Provided as a separate PDF file in the supporting submission section).

**Supporting Information 4** Figure S2: Supporting analyses of candidate gene enrichment, Mendelian randomization robustness, single‐cell clustering, and qPCR validation in chronic nonhealing wounds. (A) Circle diagram of GO biological process enrichment analysis of candidate genes. (B–E) Scatter plot, forest plot, funnel plot, and leave‐one‐out analysis for AKR1B1. (F–I) Corresponding analyses for VCAM1 demonstrating robust association with nonhealing risk. (J, K) tSNE visualization of single‐cell clustering in control and chronic nonhealing wound (CNHW) samples from the GSE265972 dataset. (L) mRNA expression levels of Beclin‐1, LC3‐II, ULK1, HIF‐1α, TNF‐α, and VEGF after QFSO treatment.  ^∗^ *p* < 0.05 versus blank group; #*p* < 0.05 versus model group; △*p* < 0.05 versus Fujifu group; ▲*p* < 0.05 versus MEBO group.

**Supporting Information 5** Figure S1: Immunohistochemical staining of PI3K, AKT, mTOR, Beclin‐1, LC3‐II, HIF‐1α and IL‐8 in the rat chronic nonhealing wound model. Immunohistochemistry results from animal experiments. (A) AKT IHC, (B) PI3K IHC, (C) mTOR IHC, (D) Beclin‐1 IHC, (E) LC3‐II IHC, (F) HIF‐1α IHC, (G) IL‐8 IHC.

## Data Availability

The transcriptome datasets analyzed in this study are available in the Gene Expression Omnibus (GEO) under accession numbers GSE174661 and GSE265972. Mendelian randomization statistics for expression quantitative trait loci (eQTL) are available from the IEU OpenGWAS project (https://gwas.mrcieu.ac.uk/). Protein quantitative trait loci (pQTL) data are from the deCODE Genetics Summary Statistics Portal (https://www.decode.com/summarydata/). Other compound target information is from the TCMSP database (https://tcmsp-e.com/tcmsp.php).
