# Supplementary figures and images for: Integrative Multiomics and Network Pharmacology Exploration of Active Components and Mechanisms of Action of Qufu Shengxin Ointment in Treating Chronic Nonhealing Wounds
Source: Mediators Inflamm. 2026 Jun 13;2026:1280142. doi: 10.1155/mi/1280142 (PMC13263708; doi:10.1155/mi/1280142)

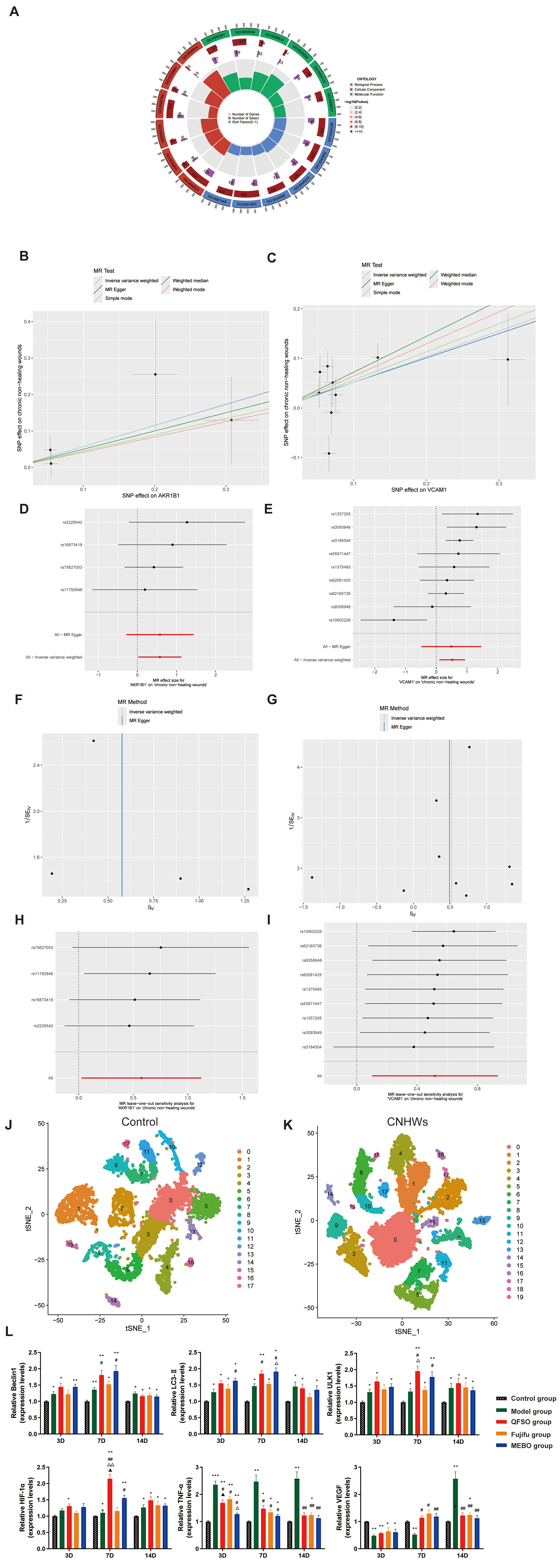

Supplement: Supplementary file 4 — Supporting Information 4 Figure S2: Supporting analyses of candidate gene enrichment, Mendelian randomization robustness, single‐cell clustering, and qPCR validation in chronic nonhealing wounds. (A) Circle diagram of GO biological process enrichment analysis of candidate genes. (B–E) Scatter plot, forest plot, funnel plot, and leave‐one‐out analysis for AKR1B1. (F–I) Corresponding analyses for VCAM1 demonstrating robust association with nonhealing risk. (J, K) tSNE visualization of single‐cell clustering in control and chronic nonhealing wound (CNHW) samples from the GSE265972 dataset. (L) mRNA expression levels of Beclin‐1, LC3‐II, ULK1, HIF‐1α, TNF‐α, and VEGF after QFSO treatment. ∗ p < 0.05 versus blank group; #p < 0.05 versus model group; △p < 0.05 versus Fujifu group; ▲p < 0.05 versus MEBO group. [file MI-2026-1280142-s004.jpg]

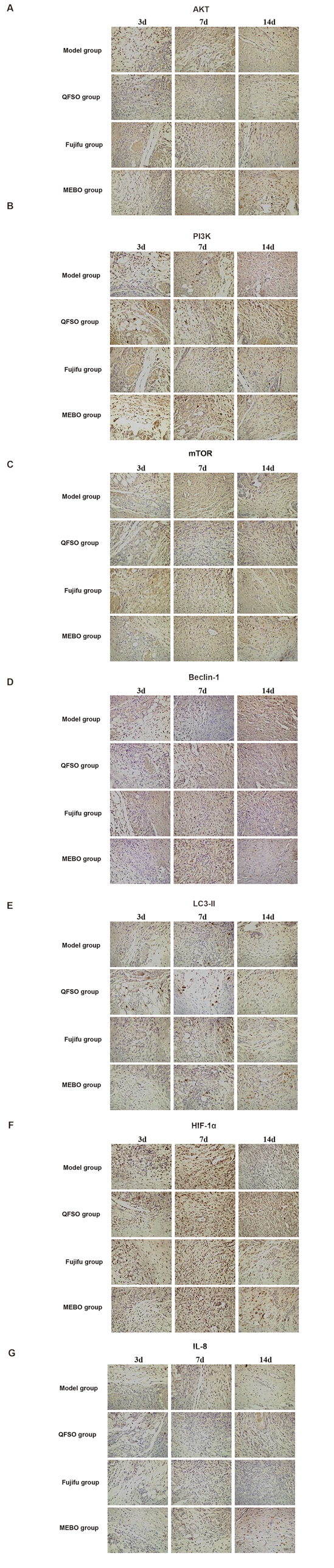

Supplement: Supplementary file 5 — Supporting Information 5 Figure S1: Immunohistochemical staining of PI3K, AKT, mTOR, Beclin‐1, LC3‐II, HIF‐1α and IL‐8 in the rat chronic nonhealing wound model. Immunohistochemistry results from animal experiments. (A) AKT IHC, (B) PI3K IHC, (C) mTOR IHC, (D) Beclin‐1 IHC, (E) LC3‐II IHC, (F) HIF‐1α IHC, (G) IL‐8 IHC. [file MI-2026-1280142-s005.jpg]
